# CRISPR-Mediated Silkworm: The Oncoming Agricultural Revolutions and a Rising Model Organism

**DOI:** 10.3390/genes17020230

**Published:** 2026-02-12

**Authors:** Qiaoling Sun, Yongkang Guo, Liting Wang, Ling Jia, Peng Wei, Sanyuan Ma

**Affiliations:** 1 Biological Science Research Center, Southwest University, Chongqing 400715, China; sunqiaoling0622@outlook.com (Q.S.); gyk666@email.swu.edu.cn (Y.G.); jialing132@163.com (L.J.); 2 Key Laboratory of Entomology and Pest Control Engineering, College of Plant Protection, Southwest University, Chongqing 400715, China; wliting01@163.com (L.W.); weipeng2019@swu.edu.cn (P.W.); 3 College of Life Sciences, Guizhou Normal University, Guiyang 550025, China

**Keywords:** CRISPR, genome editing, sericulture, silkworm

## Abstract

The silkworm (*Bombyx mori*) is essential to sericulture and is also becoming a key model organism in genomics and agriculture. For decades, genetic studies of the silkworm were limited by inefficient and inflexible genome tools. CRISPR genome editing allows precise and scalable alterations to genes regulating development, physiology, and industrial traits. This review summarizes silkworm genome-editing breakthroughs, highlighting CRISPR’s evolution from simple gene knockouts to large-scale genome-wide screening. We highlight how these advancements contribute to disease resistance, higher yields, and the development of new silk-based materials, as well as how they influence the development and growth rate of the sericulture. The creation of high-quality reference genomes, pangenomes, and genome-wide screening systems has made the silkworm a major model for integrating multiple biological datasets and approaches, such as genomic, transcriptomic, and proteomic. By considering the unique biological characteristics of the silkworm, this provides new insights for research on silk biology, piRNA synthetic biology, and hormonal signaling regulation. Finally, we examine new areas at the intersection of CRISPR, pangenomics, and artificial intelligence (AI) and suggest future paths for molecular breeding, pest control, and synthetic biology. Moreover, AI-assisted prediction of CRISPR outcomes is utilized to inform the design of targeted trait modifications, representing an approach to enhancing biomanufacturing efficiency and eco-friendly silk production. Together, these advances have made the silkworm a flexible genetic platform and an important part of sustainable agriculture and biomanufacturing.

## 1. Introduction

*Bombyx mori* is important in agriculture because it provides high-quality silk fiber, making it a valuable economic insect. Research shows it came from its wild ancestor, *Bombyx mandarina*. Around 5000 years ago, humans began domesticating and rearing this economically important species, with the selected Chinese trimolters being the earliest to differentiate from wild silkworms. As it traveled along the Silk Road, different regional strains appeared, adding to its rich diversity [1,2]. *B. mori* serves as a vital model for exploring various aspects of biology, including insect developmental processes, functional genomics, evolutionary patterns, and technological innovations [2,3,4,5]. Due to its distinctive features, such as a short life cycle, ease of large-scale rearing, and simple genetic manipulation, *B. mori* is highly valued as a silk producer. Its fibers are characterized by a lustrous appearance, soft texture, and considerable flexibility. Therefore, the species has been domesticated for silk production for millennia. Plus, their excellent mechanical strength, compatibility with living tissues, and natural ability to degrade make silk-based materials ideal for biomedical applications, such as tissue engineering, scaffolds, wound dressings, and drug delivery systems [6,7,8,9,10]. Consequently, its great application potential and advantages are establishing silkworm as an emerging model organism in insect research [3].

Understanding the intricate workings of the silkworm genome requires precise genetic techniques. Over the years, as research has deepened, technological demands have continuously evolved. Initially applied in *Caenorhabditis elegans*, RNAi technology rapidly expanded to other species, becoming a dominant tool for functional genomics in the early 21st century [11,12,13]. However, in the silkworm, RNAi via dsRNA/siRNA injection is often hindered by low success rates and incomplete knockdown [14,15]. More critically, the interference effects induced by dsRNA are transient, precluding the stable inheritance of phenotypes in subsequent generations. The subsequent emergence of gene editing technologies, including Zinc Finger Nucleases (ZFNs), Transcription Activator-Like Effector Nucleases (TALENs), and Clustered Regularly Interspaced Short Palindromic Repeats (CRISPR), has largely overcome these limitations [16]. Given the limitations of ZFNs and TALENs, such as high construction costs, complex engineering requirements, and low editing efficiency, CRISPR has become the current mainstream tool due to its simplicity, cost-effectiveness, high efficiency, and multiplex editing [16,17,18]. The emergence of the CRISPR system has significantly broken down many barriers. By transforming this natural immune mechanism found in prokaryotes into a versatile and programmable molecular tool, this technology has driven a fundamental shift—from merely disrupting genes to editing genomes with remarkable precision [19,20]. This breakthrough opens new possibilities for genetic research and therapeutic strategies [17]. Since CRISPR was successfully adapted to *B. mori* in 2013, it has really opened new avenues for genome editing in this species, enabling more efficient and precise trait modification [18,21], and exploring genome functions with remarkable ease and efficiency, driven by high-throughput methods [22,23]. The recent completion of the silkworm pangenome and T2T assemblies offers a comprehensive roadmap for functional screening [1,24,25]. This convergence enables exploration of the wide genetic diversity found in both wild and domestic strains, helping address important agricultural challenges.

This review provides an in-depth look at the latest CRISPR system and its derived tools in *B. mori*, showing how these technologies are making a big difference in five important areas: (1) industrial strain optimization, targeting economic traits such as enhanced silk yield, disease resistance, and sex-specific selection; (2) silk gland bioreactors, engineered for the efficient production of pharmaceutical proteins and recombinant spider silks; (3) fundamental biological research, uncovering molecular mechanisms underlying insect metamorphosis, physiology, and immunity; (4) genome-wide screening, employing large-scale libraries to systematically identify essential genes governing growth and stress adaptation; and (5) agricultural pest management, utilizing the silkworm as a model to develop genetic control strategies for destructive lepidopteran pests. These new advancements boost the economic prospects of sericulture. They also turn the silkworm into a sophisticated platform for synthetic biology and an essential model for tackling global agricultural challenges.

## 2. Genome Editing Technologies in Silkworm

As the main model organism for Lepidoptera and a species domesticated for thousands of years for silk production, the silkworm has always played a pivotal role in insect genetics and functional genomics [3]. The announcement of its genome sequence, along with fast-changing technologies, sparked a major boost in research and discoveries [3]. Since 2010, the introduction of programmable nucleases has truly revolutionized genome editing, leading to advances in genetic manipulation. ZFNs enabled the creation of mutations in specific genes via non-homologous end joining (NHEJ) in both somatic and germline cells. This progress has opened up many new possibilities in the field [26], while TALENs later escalated germline mutation efficiencies to approximately 50%, enabling precise site-specific knockouts [27,28]. However, the CRISPR system, noted for its simplicity, high efficiency, and capacity for multiple gene targeting, has driven major advances in genome engineering for this species [18]. These breakthroughs have substantially transformed silkworm research and enabled the development of programmable methods for genome editing. Here, we present a quick overview of how the CRISPR toolbox has evolved in *B. mori* and highlight some of its transformative applications across different biological fields.

### 2.1. From Random Mutagenesis to Programmable Genome Reshaping

In silkworms, early genetic changes mainly relied on transposon-based methods, such as the piggyBac system, making the process more accessible and reproducible [29]. These methods often show low transformation efficiency, are hard to target specific genes, and unpredictably insert DNA. The landscape began to change in the late 1990s with the emergence of the first-generation programmable nucleases, ZFNs. These advanced tools were developed by linking customizable zinc-finger DNA-binding domains to the non-specific cleavage domain of the FokI endonuclease. ZFNs operate by dimerizing at adjacent DNA half-sites, inducing a double-strand break (DSB) [30], thereby triggering repair via NHEJ or homologous recombination (HR) [31]. Although ZFNs successfully disrupted *BmBLOS2* in *B. mori*, the germline editing efficiency remained quite low, below 10%. This mainly resulted from the challenges in assembling zinc finger domains, which depend on the specific context [26].

The second-generation TALENs significantly improve upon ZFNs. Derived from a plant pathogen called *Xanthomonas*, TALENs utilize modular TALE repeats to accurately recognize a reliable RVD-based code. This design simplifies assembly and provides greater accuracy than ZFNs, improving overall efficacy and reducing technical barriers to implementation [32,33]. In 2012, TALENs marked a significant milestone in lepidopteran genome editing by successfully inducing highly efficient mutations at the *BmBLOS2* locus. This change led to a translucent appearance in the larvae, with mosaic mutation rates in the G_0_ generation reaching 27% [27]. However, the need for intricate protein engineering for each new target created technical and financial challenges, limiting their broad use in non-model organisms.

The third-generation molecular scalpel, CRISPR, originated from the adaptive immune system of bacteria [34]. It was not until 2012 that Jinek et al. revealed the key mechanism of the Type II CRISPR system, marking the emergence of a novel, programmable gene editing tool. They discovered that the catalytic activity of the Cas9 endonuclease is dependent on a dual-RNA structure formed by tracrRNA (trans-activating crRNA) and crRNA (CRISPR RNA). The resulting active complex first identifies the DNA target site via a PAM sequence (5′-NGG-3′). Subsequently, the spacer sequence hybridizes with the target strand to form an R-loop structure, driving a conformational change in Cas9 that activates its dual nuclease domains: the HNH domain cleaves the DNA strand complementary to the sgRNA (at the third nucleotide upstream of the PAM), while the RuvC-like domain cleaves the non-target strand. This process generates a blunt-ended DSB at a specific genomic locus [35]. The advent of this programmable DNA scalpel established the decisive molecular foundation for gene editing by harnessing endogenous cellular repair mechanisms, such as non-homologous end joining (NHEJ) or homology-directed repair (HDR). Applying this system to the silkworm, Wang et al. demonstrated that embryonic injection of Cas9 mRNA and two sgRNAs targeting *BmBLOS2* could induce a 3482 bp deletion. This mutation resulted in an oily-skin phenotype in 95% of the larvae, providing robust evidence of the method’s efficiency [21]. Ma et al. developed a plasmid-based system to deliver the Cas9 protein and sgRNA. This method became widely used in most subsequent genome-editing experiments. It also showed that knocking out *Bmku70*, a key part of the NHEJ pathway, could increase the rate of homologous recombination [36]. These significant early studies helped establish *B. mori* as the second insect species, after *Drosophila*, to have an established CRISPR-based genome editing system.

### 2.2. The Ever-Expanding CRISPR Toolbox: From Disruption to Precision Regulation

Since it was first developed, using the CRISPR system in *B. mori* has gone far beyond just gene disruption. Through continuous improvements, including an expanded range of targets, improved editing efficiency, and enhanced specificity, CRISPR has become a truly versatile tool for genome engineering. Today, its capabilities include not only traditional editing but also spatiotemporally controlled editing, base editing, and epigenetic remodeling (Table 1) [18].

#### 2.2.1. Canonical and Multiplex Editing

Between 2013 and 2014, the practical use of CRISPR/Cas9 in silkworms was established through knockout studies of *BmBLOS2* and *BmKu70*. In 2014, Ma et al. made a breakthrough by combining CRISPR/Cas9 with the PiggyBac transposon system, enabling efficient and heritable gene knockouts. This technology soon progressed to multiplex editing, allowing scientists to target multiple sites for large fragment deletions and simultaneous gene mutations [36,37]. In BmE cells, Ma et al. successfully created long-fragment deletions by co-transfecting a mix of gRNAs that target several sites in *BmKu70*, making the process more efficient and precise [36]. That same year, Liu et al. successfully performed targeted mutation of a single gene in the BmN cell line. Later, they further advanced the field by establishing multiplex editing of up to six genes, showcasing a major leap forward in editing efficiency [37]. Wei et al. chose the four genes with clear observable traits as targets for knockout in silkworms. The G_0_ generation displayed mutation frequencies ranging from 16.7% to 35.0%, and deletions in DNA fragments were detected [38]. Additionally, studies have also shown how the CRISPR system can be used to target RNA in silkworms, providing new perspectives for further study [39].

In addition to editing the silkworm genome, Dong et al. developed a system that specifically targets and cuts the key BmNPV replication factor, *ie-1*, thereby effectively stopping the virus from spreading [40]. To better understand how knocking out multiple sites affects efficiency, selection of various targets within the BmNPV viral genome revealed that multi-site cleavage can greatly boost the antiviral abilities of silkworms, highlighting a promising approach for improving disease resistance [41,42].

#### 2.2.2. Conditional Editing: Tissue-Specific and Inducible Systems

While constitutive knockout offers valuable insights, studying lethal genes demands more precise control. To avoid the lethality or widespread issues that come with universal knockouts, advanced tissue-specific and inducible systems have been designed. In creating these tissue-specific tools, the silkworm’s abundant promoter resources have been used to direct gene editing accurately to particular tissues. Xu et al. utilized a promoter called *nanos* (*Bmnos*), which is active in both somatic and germ cells, to ensure germ cell-specific expression. This shows that *Bmnos* is a reliable natural regulatory element in early embryos and gonads [43], and it has even been used to develop a sex-specific lethal system linked to the W-chromosome, helping to achieve effective sex control [44]. In somatic cells and the silk gland, Liu et al. used a specific *BmFibH* promoter to target *BmlaminA/C* (*BmLMN*) in the posterior silk gland (PSG). This approach led to developmental issues in the PSG and a decrease in silk production [45]. Wang et al. used the epidermis-specific *CPG25* promoter to target and disrupt the function of an epidermis-specific cuticular protein gene (*Cpr21*) and a ubiquitously expressed cuticular gene (*Cph18*), demonstrating the effectiveness of this method [46]. Additionally, Yu et al. discovered an oocyte-targeting peptide ligand in the silkworm called *BmOTP*, which is a 29-amino-acid fragment from the N terminus of silkworm vitellogenin. They attached *BmOTP* to the Cas9 protein, and by injecting this fusion protein complex into female pupae, they successfully achieved heritable gene editing in the offspring [47].

In addition to controlling space, advancements have also been made in special inducible systems, particularly those responsive to pathogens. For example, virus-inducible platforms are now available that activate Cas9 only when a pathogen invades, helping to protect against viral infections [40,41,48]. Additionally, Fang et al. combined the Cas9 protein with the secreted protein NB29-N3 from *Nosema bombycis*. This created a system that can specifically detect *N. bombycis* infection, helping to prevent its spread and proliferation [49].

#### 2.2.3. Beyond the DSB: Base Editing, Regulation, and Precise Knock-In

Beyond just disrupting genetic sequences with DSBs, the CRISPR toolkit has grown even more versatile by allowing for the tweaking of gene expression. This is performed by modifying the Cas9 protein or pairing it with other techniques. For example, Li et al. achieved precise nucleotide changes by fusing DNA encoding rAPOBEC1 with a special form of nCas9 carrying the A840H mutation, leading to the successful conversion of C to T at the *Blos2* and *Yellow-e* locations [50]. In 2020, Liu et al. compared Cas9 and BE3 (Base Editor 3) in silkworms and discovered that base editing was more effective than Cas9 in the BmE cell line: BE3 was able to introduce precise stop codons, while Cas9 could not do so [51].

Wang et al. combined catalytically inactive Cas9 (dCas9) with transcriptional repressors (KRAB, SID, SRDX) or activators (VP64 and VPR) to achieve targeted transcriptional repression or activation, respectively, in the BmE cell line [52,53,54]. Interestingly, the VPR domain showed better activation efficiencies compared to VP64. In the field of epigenetics, Liu et al. engineered a dCas9-TET1 fusion, allowing precise DNA demethylation/modification with impressive efficiencies ranging from 17.5% to 40.0% [55]. Similarly, Chen et al. developed a CRISPR–dCas9–METTL4 epigenome-editing tool that can add methyl groups to specific target regions, enabling precise m6A modifications. This helps in regulating the expression of both hypermethylated and hypomethylated genes, providing valuable insights into gene regulation mechanisms [56]. To help visualize specific endogenous genomic locations, Xing et al. created a fusion protein called NLS-dCas9-EGFP-NLS, which includes a nuclear localization signal–dead Cas9–enhanced green fluorescent protein–nuclear localization signal. By optimizing the signal-to-noise ratio in BmE cells, this tool enables the precise labeling of specific gene loci (e.g., *BmFibH*) as distinct fluorescent foci within the interphase nucleus [57]. Unlike traditional fixed-cell methods, this CRISPR imaging approach allows for the nondestructive tracking of native chromatin trajectories and spatial positioning in living cells, providing a window into the spatiotemporal dynamics of insect genomes.

Moreover, for accurate knock-in, even though it involves DSBs, it primarily utilizes mechanisms like microhomology-mediated end-joining (MMEJ) to help achieve precise integration of large DNA fragments at specific sites [58,59,60]. The spider silk protein gene was successfully integrated into the intron of the silkworm *FibH* gene using a CRISPR/Cas9-mediated gene knock-in strategy, enabling the silkworm to spin natural spider silk [61,62]. Nakade et al. used the CRIS-PITCh system to help with MMEJ, making it easier to insert external fragments into silkworms [58]. Additionally, employing single-stranded oligodeoxynucleotide (ssODN) donors to assist knock-in can effectively increase efficiency and broaden the application scope of gene knock-in techniques [63,64].

#### 2.2.4. Expanding the Scope: Variants and Toolkit Optimization

Although these applications are quite versatile, the targeting scope was initially limited by PAM constraints. However, progress has been made by expanding the editable sites through the development of variants like xCas9 and Cas9-NG [65]. Meanwhile, other nucleases like SaCas9 and Cpf1 (Cas12a) have also been explored, along with their respective PAMs (5′-NNGRRT-3′, 5′-TTN-3′) [66,67]. In addition to expanding PAM sites, efforts have also been made to optimize different components. For instance, they have improved the sgRNA scaffold and chosen better promoters, like modifications using U6 or T7 promoters, to make the process even more efficient [68,69], and optimized the codon of Cas9 to improve genome editing efficiency [63]. Delivery methods also received special attention. RNP (ribonucleoprotein) complex injection techniques were crafted that, compared to plasmid injection, not only made the process more efficient but also helped minimize off-target effects [70].

As more data was gathered, evidence indicated interesting patterns in editing results. It has been found that the patterns of CRISPR/Cas9 indels vary between different species. For example, in silkworms, indels mostly happen upstream of the cut site, while in zebrafish (*Danio rerio*), they mainly occur downstream. Additionally, deletions mediated by proximal microhomologous sequences represent the most frequent mutation type [22]. Deep sequencing has shown that the Cas9 mutation patterns in silkworms are consistent and can be anticipated. It also revealed that insertions and deletions are not entirely random; instead, they often involve microhomology-mediated deletions. This insight offers a helpful foundation for designing more precise experiments [71].

**Table 1:** Summary of advanced CRISPR/Cas9-derived tools and their applications in *Bombyx mori*.

Ref.	Study	CRISPR Tool/System	Target/Strategy	Performance in *B. mori*	Key Applications	Limitations
[21]	Wang et al., 2013	Standard SpCas9	Knockout (Indel) DSB-mediated NHEJ	High (94.0–95.6%)	Gene disruption; Large fragment deletion (~3.5 kb); Proof-of-concept (e.g., *BmBLOS2*).	Random Indels; Unpredictable outcomes; Frameshift mutations.
[36,38]	Ma et al., 2014; Wei et al., 2014	Multiplex/Heritable Cas9	Multiplex Editing; PiggyBac/ multi-gRNA	16.7–35.0% (G0 stage); Heritable mutagenesis achieved	Heritable site-directed mutagenesis (e.g., *BmKu70*); Multiplex genome editing (up to 3 sites/gene).	Variable gRNA efficiency: Lower efficiency in embryos compared to cells.
[43,45,47]	Xu et al., 2019; Liu et al., 2017; Yu et al., 2023	Tissue- Specific Cas9	Conditional Editing Promoter-driven (*Bmnos*, *BmFibH*, *CPG25*) or Ligand fusion (BmOTP)	Restricted expression to target tissues (Germline, Silk gland, Epidermis)	Tissue-specific control; Sex-control systems (W-linked); Silk yield modification (*BmLMN*); Oocyte-targeted editing.	Promoter leakage; Limited availability of strictly tissue-specific promoters.
[40,49]	Dong et al., 2016; Fang et al., 2024	Inducible Cas9	Inducible Editing Pathogen-responsive complex	High antiviral activity; Triggered by infection	Smart defense systems; Inhibiting BmNPV replication (*ie-1*); Detecting infection of *Nosema bombycis*.	Response latency; Dependent on pathogen load for activation.
[50,51]	Li et al., 2018; Liu et al., 2020	Base Editor (BE3)	Base Editing C-G to T-A conversion	40–53.75%	Precise point mutation; Creating premature stop codons; Correcting SNPs without DSB.	Off-target effects (DNA/RNA); Restricted by PAM and editing window.
[58,59]	Nakade et al., 2014; Wan et al., 2024	CRIS-PITCh/MMEJ	Knock-in MMEJ-mediated	Variable/High (Locus-dependent)	Targeted integration; Inserting fluorescent markers (e.g., 3xP3-DsRed2 into *Fib-L*).	Lower efficiency than NHEJ; Requires construction of microhomology vectors.
[52,54]	Wang et al., 2019; Hu et al., 2021	CRISPRa	Activation Transcriptional regulation dCas9-VP64/VPR	Up to 8000-fold	Gain-of-function; Upregulating silk proteins (*BmFib-H*) in BmE.	Context-dependent; potential toxicity of VPR domain; Transient effect.
[53]	Wang et al., 2019	CRISPRi	Repression dCas9-KRAB/SID/ SRDX	Significant repression	Gene silencing; Loss-of-function analysis without DNA damage; Alternative to RNAi.	Incomplete knockdown: Transient effect compared to stable KO.
[55]	Liu et al., 2019	dCas9-TET1	Epigenetic Editing Demethylation	17.5–40.0%	Epigenetic regulation; Modifying DNA methylation status to study gene regulation.	Incomplete conversion: Effect may not be heritable or stable.
[56]	Chen et al., 2024	dCas9-METTL4	Epigenetic Editing 6mA Modification	Detected (Qualitative)	N6-methyladenine editing; Site-specific installation of 6mA (e.g., proteasome subunit).	Novel tool; Functional consequences of 6mA still under investigation.
[23,72]	Ma et al., 2024; Chang et al., 2020	CRISPR Library	Screening Genome-wide KO	1726 lines (High throughput)	Forward genetics; Identification of essential genes, toxin resistance, and visible phenotypes.	Labor intensive; Requires large-scale rearing and screening infrastructure.
[67]	Zou et al., 2025	Cas12a (Cpf1)	Knockout T-rich PAM	Lower than Cas9	AT-rich targets; Alternative to Cas9 for T-rich genomic regions; RNP delivery.	Lower efficiency compared to Cas9; Temperature sensitivity.
[57]	Xing et al., 2020	CRISPR Imaging	Visualization dCas9-EGFP	Visualized	Live imaging; Tracking endogenous genomic loci dynamics in living cells.	Signal-to-noise ratio; non-editing application only.

## 3. Agricultural Revolutions: Modernizing Sericulture

### 3.1. Addressing Long-Standing Challenges in Sericulture

**Figure 1:**
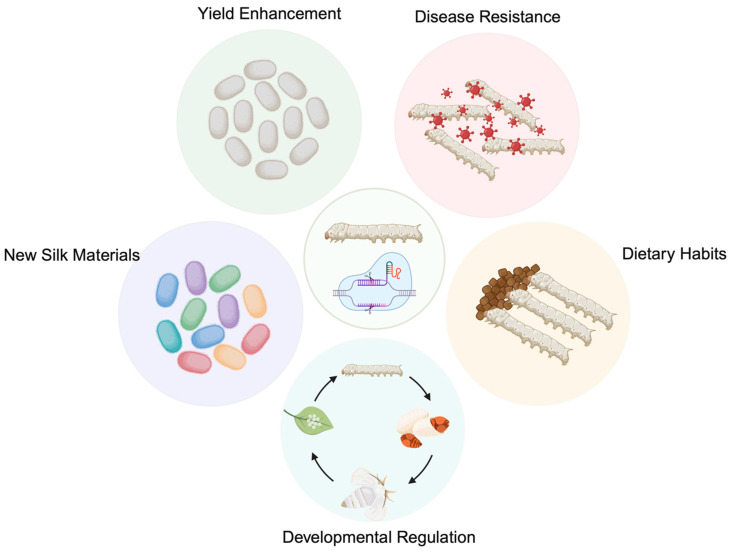
CRISPR-mediated precision breeding as a central engine for sericultural modernization. Instead of relying on traditional selection, targeted genome editing enables the simultaneous optimization of production traits (yield and developmental rate), physiological robustness (disease resistance and dietary adaptability), and product diversification (novel silk materials), thereby transforming the silkworm into a highly efficient and versatile biofactory.

For millennia, sericulture breeding has strived to optimize key economic traits, including silk yield, disease resistance, and developmental timing [73,74,75], as well as to develop specialized strains such as male-only populations and “permanent cocoon” lines [76,77]. While traditional hybridization utilizing natural germplasm resources has ameliorated some issues—such as viral susceptibility—many breeding objectives remain difficult to achieve through classical methods alone. The advent of the CRISPR/Cas9 system has provided a significant opportunity to overcome these challenges (Figure 1).

#### 3.1.1. Yield Enhancement and Developmental Regulation

Increasing silk yield has consistently remained the central objective for maximizing economic returns in sericulture. While traditional hybrid breeding has yielded results, it is constrained by long breeding cycles, unstable traits, and high labor intensity, making it difficult to sustainably select superior strains that possess both high yield and stability [73]. In recent years, CRISPR/Cas9 technology has overcome these problems by editing factors related to growth and development, as well as components in hormone signaling pathways.

Studies by Wang et al. demonstrated that the loss of function of miRNA let-7 promotes endoreplication in silk gland cells, resulting in enlarged posterior silk glands and a concomitant increase in silk yield, although such gains are strictly contingent upon tissue-specific targeting to avoid developmental trade-offs [78,79]. Furthermore, systemic knockout of let-7 was found to promote systemic larval growth and significantly increase silk yield by approximately 50% [80]. Regarding kinase and metabolic regulation, Li et al. discovered that knocking out *BmEcKL1*, a member of the Ecdysteroid kinase-like family (EcKLs), in the middle and posterior silk glands increased the weight of these glands by approximately 10% and 30%, respectively, leading to increased cocoon weight and silk fiber density [81]. Additionally, Wu et al. identified a previously unannotated negative regulator in insects—SGDAcn. Their research revealed that its knockout relieves the inhibition of the EGFR/PI3K/AKT and SGDAcn-NF-κB pathways, thereby promoting endoreplication and cell growth in silk gland cells. This knockout also enhances ribosome biogenesis and protein synthesis within the silk gland, resulting in an approximate 20% increase in the cocoon shell rate of male silkworms [82]. Extensive loss-of-function studies have also elucidated the essentiality of silk protein-related components, signaling pathway constituents, key transcription factors, and metabolic genes for silk gland development [1,83,84,85,86,87].

In addition, regulating developmental duration is also a pivotal strategy for balancing silk yield with production efficiency. Utilizing the CRISPR system to modulate the larval-to-pupal transition typically involves either extending the 5th instar developmental period to augment silk protein accumulation or shortening the life cycle to improve rearing turnover rates. Knockout of the juvenile hormone-related genes *BmJHE* and let-7 prolonged the development time of the last instar larva, thereby achieving a significant increase in cocoon yield [80,88]. Moreover, mutations in the circadian gene *Clock* can prolong larval development, resulting in an approximate 7% increase in silk yield and a 25% increase in pupal weight compared to the wild type [89]. Conversely, Cao et al. demonstrated the effects of shortening developmental duration. Knockout of *Bmdimm* accelerated development from the 3rd instar onwards, advancing the wandering stage by 2.5 days compared to the wild type, which consequently led to thinner cocoon shells [90]. Although this phenotype is currently associated with reduced silk yield, this finding offers important insights for future breeding: specifically, how to maintain cocoon shell volume while shortening the rearing cycle to cultivate “fast-growing, high-yielding” strains.

Given that male silkworms exhibit better traits regarding cocoon shell ratio, quality, and lower food consumption compared to females [44,91,92], the establishment of all-male strains is a critical direction for enhancing industrial efficiency. In 2018, Zhang et al. developed a female-specific embryonic lethality binary system based on TALEN and CRISPR/Cas9 technologies. One strain expresses Cas9 on the W chromosome driven by the germline-specific promoter nanos, while the other utilizes the U6 promoter to drive sgRNA targeting transformer 2 (*tra2*), a key gene regulating sex determination and embryonic development. The results showed that in the F1 hybrid progeny, approximately 50% of the embryos (the females) died, while the male individuals survived normally to adulthood [44].

Additionally, addressing the post-harvest processing stage, sericulturists typically must employ high-temperature or freezing treatments to kill pupae to prevent moth eclosion and the secretion of cocoonase, which damages the silk. To resolve this issue, Gai et al. created a “permanent cocoon” by knocking out *Cocoonase*. The resulting mutant moths undergo normal eclosion but are unable to secrete the enzyme required to dissolve the silk, subsequently dying inside the cocoon [77]. This innovation significantly reduces energy consumption and labor costs.

#### 3.1.2. Disease Resistance

As a fully domesticated economic insect, the silkworm has undergone directional selection to achieve efficient silk production. However, high-density rearing environments promote the spread of pathogens, where individual infections can rapidly escalate into devastating population-wide epidemics, causing severe economic losses. Currently, three major categories of pathogenic microorganisms—bacteria, viruses, and fungi—pose serious threats to sericulture, as these diseases not only lead to mass mortality but also significantly reduce the yield and quality of cocoons [93,94]. To address disease challenges, CRISPR-based gene editing technology has been utilized to perform precise single-target or multi-target editing on the silkworm host or pathogen genomes, successfully creating highly resistant germplasm resources.

Regarding defense against bacterial infection, research has focused on editing key innate immunity genes to bolster the host’s defensive capabilities. Ye et al. discovered that knocking out the silk protein gene *Sericin1* induces the upregulation of multiple immune-related genes (such as antimicrobial peptides) and protease inhibitors to maintain the immune homeostasis of silk gland cells [95]. KPI5 serves as a key negative regulator of immunity. Studies have shown that although *KPI5* mutation inhibits the prophenoloxidase activation pathway in the hemolymph, it simultaneously promotes the expression of antimicrobial peptides, revealing its dual function in regulating innate immune homeostasis [96]. The Toll signaling pathway, as the core of antibacterial defense, is also negatively regulated by serine protease inhibitors. Liu et al. elucidated that serine protease inhibitors *Serpin-1a* and *Serpin-6* inhibit the Toll pathway by suppressing the key enzyme CLIP2, which is responsible for processing Spätzle into its active ligand form. Therefore, knocking out these genes can relieve the inhibition, thereby activating the Toll pathway and enhancing the antibacterial ability of silkworms [97]. Additionally, Xiong et al. confirmed that phospholipase A2 (*BmsPLA2-4*) is an important component of the midgut immune barrier [98]. The immunoglobulin superfamily member *BmHemolin* can promote hemocyte aggregation and nodule formation, accelerating the encapsulation and clearance of pathogens [99].

To enhance antiviral capabilities, many studies have used BmNPV as a model, disrupting key viral replication genes to block its proliferation. Early work primarily targeted single viral genes. Dong et al. constructed an inducible knockout system targeting the essential viral replication gene *ie-1* in BmN cells [40] and subsequently achieved breakthroughs in practical applications [100]. Knocking out the viral anti-apoptotic gene *iap2* inhibits mitochondrial oxidative phosphorylation and activates host cell apoptosis, accelerating the apoptosis of infected cells to limit viral proliferation [93,101]. Additionally, mutations in the *orf76* gene have been proven to be an effective method for blocking BmNPV infection [102]. Notably, to expand the library of BmNPV resistance targets, Chang et al. established the first genome-wide CRISPR/Cas9 knockout library in BmE cells. This high-throughput screening platform enabled the identification and validation of a batch of key BmNPV resistance genes [72]. In addition, multi-site editing strategies have been implemented to reduce the risk of viral escape via point mutations. By targeting multiple viral genes (*ie-1* and *me53*, *lef-1* and *lef-3*), they significantly inhibited the replication and proliferation of BmNPV, and the resistance can be inherited by offspring [41,103]. Liao et al. edited multiple sites of *ie-1* and found that it could more thoroughly knock out the viral gene and hinder its spread [104]. Additionally, Liu et al. pioneered a “multi-target” cleavage strategy. By designing a single sgRNA targeting widely distributed homologous repeat sequences in BmNPV, it leads to large-scale breaks and deletions in viral DNA, thereby inhibiting its proliferation [42].

Beyond directly targeting viral genes, editing host susceptibility and resistance genes constitutes another potent defense strategy. Liang et al. demonstrated that the midgut membrane protein BmSUH interacts directly with the viral ODV-E66 protein, mediating the adhesion and entry of virions into the midgut epithelium; thus, BmSUH serves as a potential target for blocking viral invasion [105]. Conversely, the serine protease BmSPP directly inhibits viral proliferation by activating the prophenoloxidase system to promote hemolymph melanization [106]. Epigenetic research shows that the N6-methyladenosine (m6A)-related methyltransferases METTL3 and METTL14 are upregulated in expression during BmNPV infection, suggesting that the m6A modification pathway can serve as a potential target for antiviral engineering [107].

Microsporidia represent another critical pathogen causing yield decline. Dong et al. developed an *HSP70* promoter-driven inducible CRISPR/Cas9 system to confer resistance. Activated upon infection, this system efficiently edits the host gene *BmATAD3A*, significantly inhibiting the expression of key microsporidian genes and improving silkworm survival without affecting host development [108]. Regarding immune signaling, STING (Stimulator of Interferon Genes) is a key adaptor in innate immunity that also participates in microsporidian infection. Hua et al. found that *BmSTING* knockout silkworms exhibited significantly lower morbidity during early infection compared to the wild type [109]. Furthermore, Ran et al. elucidated the mechanism by which microsporidia inhibit host apoptosis: the secreted serine protease inhibitor NbSPN14 binds directly to the silkworm Caspase homolog BmICE, blocking its nuclear translocation. CRISPR-mediated knockout of *BmICE* or mutation of the *NbSPN14* P1 site abolished this anti-apoptotic effect, providing a target to block pathogen proliferation [110]. Fang et al. established a host-mediated inducible CRISPR/Cas9 system. By fusing Cas9 with the microsporidian secretion protein NB29-N3, the fusion protein is transported into the pathogen upon infection or *NB29* overexpression, enabling target gene editing [49]. This breakthrough opens new pathways for direct genomic intervention in microsporidia. Besides microsporidia, *Beauveria bassiana* is also an important pathogen that harms silkworms. It has been revealed that signal peptide peptidase SPP is a key defense factor and confirms the core position of SPP in the antifungal immune defense network [111].

### 3.2. Expanding the Frontiers of Sericulture: Novel Materials and Dietary Adaptation

The advent of CRISPR/Cas9 has revolutionized the sericulture industry, extending its scope far beyond traditional breeding objectives like high yield and disease resistance. Current research leverages this technology to conquer two new frontiers. One approach involves capitalizing on the natural compartmentalization of the silk gland to engineer novel silk-based materials, most notably recombinant spider silk and biomedical proteins. Another strategy focuses on modifying metabolic pathways to facilitate dietary adaptation, thereby optimizing silkworms for cost-effective rearing on artificial diets (Figure 1).

#### 3.2.1. New Silk Materials

The silkworm silk gland exhibits exceptional silk protein synthesis capability, enabling efficient secretion of large quantities of fibroin and sericin proteins, thereby supplanting other silk-producing animals as an ideal model for green biomaterial research [112]. Leveraging this unique feature, genetic engineering techniques have been creatively applied to precisely integrate exogenous genes into different regions of the silk gland, thus efficiently producing recombinant proteins with distinctive functions.

There are three main strategies for recombinant protein production that leverage the tissue specificity of silk glands. The first strategy involves producing pharmaceutical proteins. As biofactories, silkworm glands are utilized to synthesize high-value therapeutic proteins, ranging from human collagens (types I–III) and growth factors (hEGF, bFGF) to interferons and specialized cuticle proteins [6,113,114,115,116,117,118]. Concurrently, research aimed at augmenting the mechanical properties of silk fibers has achieved significant improvements in toughness and strength by incorporating structural proteins—such as spider dragline silk proteins(MaSp), *Drosophila* resilin, and the glycoprotein dumpy—as well as molecular modifiers like artificial amino acids (3-AzTyr), bond-forming proteins (BFAPs), and metal ions (Fe^3+^) [61,119,120,121]. The third category highlights functional modification, such as incorporating fluorescent proteins like GFP and RFP into fibroin to create naturally colored silk. Unlike traditional dyeing methods or pigment expression in sericin—which can fade during degumming—colors embedded directly into the fibroin backbone are highly stable. This approach not only reduces chemical pollution but also helps conserve water resources [122].

Among the many exogenous proteins, spider silk stands out because of its incredible strength, remarkable toughness, and lightness. When comparing weight for weight, its mechanical properties surpass those of steel and Kevlar, establishing it as one of the most robust natural biological materials [123]. However, because spiders tend to be territorial and sometimes even cannibalistic, they are difficult to domesticate. This makes it important to find suitable alternative hosts that can be used for large-scale production of recombinant spider silk proteins [4]. The silkworm is a cost-effective choice as a bioreactor platform for producing recombinant proteins [124]. In 2018, Xu et al. successfully addressed the challenge of low expression levels seen with exogenous gene knock-ins. They used a TALEN-mediated method to replace Fib-H with the spider *MaSp1* gene; this approach dramatically improved the expression efficiency of the foreign protein, increasing it from just 2–5% to an impressive 35.2% [4,7]. That same year, You et al. demonstrated that various repetitive motifs in the primary structure of the spider silk protein MaSp1 determine the mechanical properties of the fiber [125]. In 2019, Zhang et al. created high-performance spider silk fibers with the help of the CRISPR/Cas9 system. By using a specially modified CRISPR system, they carefully inserted a natural, ultra-long spider silk gene (about 10 kb) into the intron regions of the silkworm’s fibroin heavy and light chain genes. The resulting transgenic silk showed mechanical strength on par with natural spider silk, which is quite impressive. Plus, because the foreign gene was inserted into non-coding regions, it did not disrupt the inheritance of other genes in the silkworm [62]. Subsequently, Mi et al. completely replaced the repetitive region within the silkworm fibroin component Fib-H with MiSp, enabling transgenic silkworms to express and spin full-length spider silk protein. Compared to industrial materials such as nylon and Kevlar, this novel silk demonstrated exceptional tensile strength (1299 MPa) and ultra-high toughness (319 MJ/m^3^). This study overcame the previous technical bottleneck regarding the knock-in of full-length spider silk genes and successfully resolved the structural dilemma of balancing high strength with high toughness in recombinant silk fibers [61].

#### 3.2.2. Modification of Dietary Habits and Large-Scale Breeding

The silkworm is a representative example of an oligophagous insect that depends entirely on mulberry leaves in traditional sericulture. While the nutritional makeup of mulberry leaves is well-suited to support silkworm growth and is generally affordable, the seasonal nature of mulberry tree growth means that breeding is limited to spring and autumn, making year-round production challenging. Additionally, cultivating mulberries in open fields can expose the plants to pathogens and pesticide drift. Interestingly, leaves picked during rainy seasons often have excess moisture, which can make silkworms more vulnerable to bacterial and fungal infections, leading to significant economic setbacks [126,127,128,129,130]. To address issues like resource seasonality and safety concerns, expanding the silkworms’ diet options through gene editing and creating affordable, versatile artificial diets are important steps forward. These innovations are key to modernizing and growing the sericulture industry, making it more efficient and reliable.

*cis*-jasmone, a key volatile compound found in mulberry leaves, is crucial in triggering the silkworm’s feeding response. Studies support that removing the insect-specific odorant receptor co-receptor (Orco) hampers the larvae’s sense of smell, leading to a reduced ability to identify and choose mulberry leaves or *cis*-jasmone specifically [131]. Regarding specific receptors, *BmOR56* has been identified as an olfactory receptor. Although knocking out *BmOR56* did not significantly change feeding on mulberry leaves, it caused a noticeable drop in the intake of artificial diets, showing a reliance on the amount of mulberry powder [132]. Gene editing focusing on gustatory receptors (GRs) has shown impressive results. Zhang et al. found that mutations in the taste receptor BmGr66 can directly influence silkworm feeding preferences. Mutant larvae were observed to feed on Mongolian oak leaves (*Quercus mongolica*), apples, pears, soybeans, and corn, and they experienced notable weight gain when consuming fruits and grains [133]. Similarly, Endo et al. showed that the deletion of *BmGr6* and *BmGr9*—both part of the GR family—leads to a loss of host recognition ability. Further molecular analysis indicated that this change is due to a significant decrease in sensitivity to two important secondary metabolites in mulberry leaves: chlorogenic acid (CGA) and isoquercitrin (ISQ), which results in the loss of feeding preference [134]. Meanwhile, subsequent findings have further explored the GR family. It has been shown that several different GRs—such as *Gr15*, *Gr29*, *Gr43*, and *Gr66*—play a role in shaping the oligophagy behavior of silkworm larvae [135,136]. Interference with the transcription factor Zfh3 alters both gustatory and olfactory functions in silkworms. This interference removes their single-food preference for mulberry leaves, allowing larvae to try a wider variety of foods and boosting their ability to adapt to artificial diets without mulberry components [137].

## 4. CRISPR-Driven Elevation of the Silkworm as a Model Organism

Harnessing detailed data from the silkworm’s rich genome, CRISPR/Cas9 and related tools are used to probe its unique biological features, such as silk production linked to silk glands, piRNA biosynthesis, and hormonal networks involved in insect metamorphosis. By combining large-scale functional screening, such as CRISPR libraries, silkworm gene function can be more effectively validated and analyzed, thereby deepening the understanding of the silkworm’s genome. This effort has transformed the silkworm from a traditional economic insect into a versatile model organism that integrates multi-omics and systems biology.

### 4.1. Comprehensive Genomics and Multi-Omics Resources

Comprehensive and detailed genomic data, coupled with abundant multi-omics resources, have significantly cemented *B. mori*’s status as a model organism for Lepidoptera, while also serving as the foundation for CRISPR-mediated genome modification (e.g., precise editing) and functional studies (e.g., high-throughput screening). Since the initial release of the silkworm genome in 2004, the assembly has undergone multiple iterations of refinement. The early draft covered approximately 97% of the genome, unveiling a landscape rich in repetitive sequences and transposon-related structures [3]. After that, with improvements in sequencing technology, more complex genomes were assembled. Among them, the most used genome databases are Kaikobase, Silkbase, and SilkDB3.0 [138,139,140]. Most recently, to resolve complex genomic regions such as telomeres, rDNA clusters, and tandem repeats that remained elusive in previous assemblies, the gap-free T2T genome (without the W chromosome) was attempted to be assembled [24,25]. At the same time, the W chromosome also has a refined assembly [141]. To transcend the limitations of a single reference genome in capturing genetic diversity, Tong et al. conducted deep re-sequencing of 1078 silkworm strains and assembled 545 long-read genomes to construct a high-resolution pangenome map [1]. Beyond genomics, the multi-omics landscape has been further enriched by the development of integrated databases such as SGID and the organelle proteome database SilkOrganPDB, as well as the creation of the first high-throughput SNP array, Silk_40K [142,143,144].

### 4.2. Unique Biological Characteristics and Mechanisms

Given the biological uniqueness of the silkworm, such as the silk gland functioning as a highly efficient protein factory, the unique piRNA mechanism in sex determination, and the clear stage-specific regulation of hormonal signaling, this section will elaborate on the role of CRISPR in these areas and its systematic advances.

#### 4.2.1. Silk Biology

Although studies have shown that at least 140,000 species have been confirmed to secrete silk proteins [112], the silkworm serves as an exemplary species in which precise genome editing has been investigated and well-established. In the realm of silk biology, the CRISPR tools have facilitated the decoding of the molecular machinery of silk synthesis and organ development. For instance, the precise coordination of silk structural proteins has been elucidated by knockout studies targeting the fibroin heavy chain (*Fib-H*) and fibroin light chain (*Fib-L*); deficiencies in these genes compromise the integrity of the PSG and result in naked pupae or thin cocoons, highlighting their essential role in preventing premature gland degradation [145,146]. Furthermore, although the Fhx protein is not essential and its loss results only in modest changes in silk quantity, quality, and composition, the integrity of the secretory pathway nevertheless relies on auxiliary proteins, as evidenced by the pronounced reorganization of the rough endoplasmic reticulum following mutation of the *Fhx* gene [83,147]. More recent investigations have identified novel components such as fhx-L1, which influences the structural assembly and β-sheet content of the silk fiber [148], and BmSuc1, an animal-type β-fructofuranosidase (β-FFase) that modulates silk properties by regulating *Ser1* expression in the middle silk gland [149].

Despite current progress in understanding silk gland development and fiber assembly [112,150], the molecular mechanisms driving this ultra-efficient translational machinery remain largely obscure. CRISPR-mediated multiplex gene editing offers an opportunity to systematically dissect the key regulatory factors and genetic networks governing this massive protein output. This could facilitate deciphering the determinants of translational efficiency, thereby optimizing the silk gland as a high-efficiency biofactory for exogenous protein production.

#### 4.2.2. piRNA Biology

Another relatively unique biological characteristic of the silkworm is piRNA-based sex regulation [151]. What makes the silkworm an excellent model for piRNA research is not only this simple, linear, and causally well-defined piRNA sex-determination mechanism, but also the availability of the BmN cell line, a cell line that can normally synthesize piRNAs [152]. Using CRISPR/Cas9 technology, studies have found that, for sex determination in the silkworm, SIWI and its cofactor BmAsh2 play indispensable roles, and that *BmSxl* and *BmPnldc1* are essential for spermatogenesis; in contrast, the factors BmAgo3 and BmMael appear to be nonessential in the sex-determination process [153,154,155].

Regarding the silkworm piRNA pathway, recent studies using BmN4 cells revealed that pre-piRNAs are generated via two parallel endonucleolytic mechanisms: Zucchini (BmZuc)-mediated cleavage, which recognizes specific consensus motifs on Siwi-loaded precursors, and PIWI-catalyzed slicing [156]. Furthermore, a mitochondria-associated endoribonuclease, BmRNase κ, was identified to promote robust piRNA production by generating 2′,3′-cyclic phosphate-containing precursors [157]. Following these cleavage events, the 3′ ends of pre-piRNAs are matured by the exonuclease Trimmer (*PNLDC1*), a process recruited by the Tudor domain protein Papi and coupled with 2′-O-methylation [152,156]. Other auxiliary factors, such as *BmGTSF1*, which interacts with *BmSIWI* [155], and *BmQin*, which localizes to the nuage [158], are also integral to maintaining the stability and function of this pathway.

The piRNA pathway not only dominates sex determination but also participates in transposon silencing, genome defense, and antiviral processes [159,160,161]. Although numerous factors involved in piRNA transcription, transport, and processing have been identified [162,163], there are still gaps in understanding of this pathway in terms of transgenerational inheritance, evolutionary adaptability, and interspecies differences. Leveraging silkworm cell lines that retain a complete piRNA metabolic network, combined with CRISPR-based high-throughput screening, offers a chance to further dissect these complex mechanisms.

#### 4.2.3. Hormone Signaling

As a holometabolous insect, the silkworm has a clearly demarcated developmental timeline that provides an excellent temporal window for precisely capturing hormone-induced physiological transitions. The molting hormone, 20-hydroxyecdysone (20E), acts as a key signaling molecule for metamorphosis, binding to the canonical nuclear receptor complex EcR/USP and functioning in the nucleus. Once induced by 20E signaling, its downstream primary transcription factors further regulate the expression of secondary transcription factors, thereby initiating larval molting from one instar to the next as well as metamorphic molting from larva to adult [164]. Using the CRISPR/Cas9 gene-editing system, the roles of the *BmEO* and *BmHR38* genes in the 20E regulatory network are clearly analyzed [165,166]. Juvenile hormone (JH) antagonizes 20E to maintain the larval phenotype [167]. To further elucidate JH signaling and for functional validation, using CRISPR, knockout models of *BmJHE*, *BmFOXO*, and *Met1* were used to analyze the roles of these genes in the JH signaling regulatory pathway [88,168,169].

Hormone signaling transduction in the silkworm is not a simple linear process. Instead, it functions as a highly integrated complex network of interactions involving developmental, metabolic, and environmental signals [170]. In this context, CRISPR-mediated conditional gene knockouts and activation technologies endow the capability to achieve precise targeted editing under specific conditions, promising to reveal the core genetic mechanisms that dominate developmental fate. Furthermore, the application of CRISPR high-throughput screening will assist in discovering unknown key regulatory factors, thereby systematically completing the overall framework of these signaling regulatory networks.

### 4.3. Genome-Wide Screening and Libraries

Genome-wide CRISPR screening is a vital tool in functional genomics, widely used in mammalian cell lines, and has led to breakthroughs. However, in non-model multicellular organisms, especially insects, progress has been hampered by challenges such as creating effective sgRNA libraries, ensuring stable transfection, and conducting in vivo physiological validation [22]. Recently, the development of CRISPR screening tools in silkworms—from studying cells to creating large libraries of mutants—has truly pushed this field into a new, high-speed stage of discovery [23].

#### 4.3.1. Cell-Based High-Throughput Screening

The main difficulty in establishing genome-wide screening in non-mammalian animals is the lack of systems capable of supporting large-scale, high-throughput lentiviral library delivery [72]. Silkworm cell lines (such as BmE and BmN), which are easy to culture and transfect, have become ideal platforms for overcoming the technical challenges of genome-wide screening. Chang et al. were the first to construct a CRISPR/Cas9 knockout library covering the whole genome and performed screening in BmE cells, identifying 1006 genes essential for cell survival [72]. They further revealed the critical roles of steroid and fatty acid biosynthesis pathways under temperature stress, as well as 1614 candidate genes associated with BmNPV infection, 10 of which were experimentally validated. This library has subsequently been widely used in studies of responses to environmental toxicants: Liu et al. identified hundreds of genes mediating the toxicity of sodium fluoride and cadmium chloride, elucidating the common or specific roles of MAPK signaling, DNA repair, and the Toll/Imd and autophagy pathways [171].

Further expanded applications include the regulation of hormone signaling and pH homeostasis. Sun et al. used this library in combination with 20E treatment to directly screen and identify 324 20E-responsive genes, which are mainly localized in the nucleus and cytoplasm, revealing that 20E signaling coordinates physiological processes through multiple pathways such as phosphorylation, innate immunity, metabolic homeostasis, and apoptosis [172]. Recently, Chang et al. discovered, through screening, that multiple genes regulate intracellular pH homeostasis, highlighting the powerful potential of the library for dissecting cellular physiological mechanisms [173]. In addition, Sun et al. conducted a screen targeting the juvenile hormone signaling pathway, further enriching the comprehension of hormone regulatory networks [174]. Together, these studies demonstrate that cell-level genome-wide screening has become an efficient engine for functional genomics research in the silkworm and has laid the foundation for subsequent in vivo validation.

#### 4.3.2. Large-Scale Mutant Library Creation and In Vivo Screening

Compared to cell-based screening, in vivo functional validation offers a more accurate reflection of physiological conditions. Traditional single-gene knockout methods are often time-consuming and inefficient, hindering large-scale studies; thus, the development of large-scale mutant libraries is regarded as a breakthrough. These libraries allow pooled screening, enable parallel functional analysis, and serve as valuable resources for studying complex genetic traits in a more accessible and efficient way.

Ma et al. have successfully built a comprehensive mutation library for the silkworm. They designed an impressive 92,917 sgRNAs that target promoter and exon regions across 14,645 protein-coding genes. Additionally, they established 1726 transgenic sgRNA lines and obtained 300 stable mutant lines, demonstrating a range of visible traits and economic benefits. Using CRISPR-based pooled mutant library screening for cadmium tolerance, *KWMTBOMO12902* was identified as a potent candidate gene for sericultural breeding applications. Notably, knockout of this gene confers resistance to cadmium [23].

Meanwhile, large-scale genome editing produces extensive sequencing data, opening new opportunities to better understand how CRISPR-Cas9 works in insects. By studying large mutant libraries, researchers have discovered that in silkworms, deletions are the most common result, accounting for 86.50%. These deletions tend to happen mainly in one direction, mostly upstream of the cut site, which aligns with the team’s earlier exploratory findings [22,71]. The study also found that the GC content of the sgRNA sequence, especially nucleotides 2–10 in the PAM-distal region, tends to correlate with higher editing efficiency. Additionally, epigenetic chromatin modifications, such as 6mA methylation, can boost editing success. These findings, based on extensive data, not only help elucidate DNA repair processes in insects but also offer valuable guidance for developing more effective and accurate genome-editing tools for silkworms in the future.

## 5. Future Directions and Perspectives

**Figure 2:**
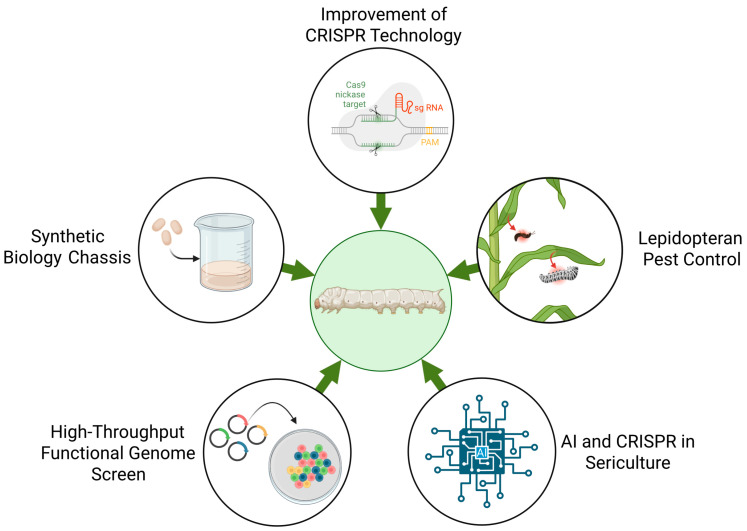
Future directions and perspectives in silkworm. The development directions of the CRISPR system in the silkworm mainly include further enhancing the application of CRISPR technology in silkworms, transforming silkworms into a biosynthesis platform, expanding its use in the control of lepidopteran pests, employing it for genome-wide screening, and integrating it with AI.

The arrival of the CRISPR system has propelled sericultural science, transforming the silkworm from a traditional silk producer into a genetically tractable model organism. While the first decade of CRISPR use helped establish essential editing techniques and uncovered important developmental pathways, we are now moving toward more precise, high-throughput, and industrial-scale applications. We are also seeing the silkworm become a versatile platform for synthetic biology—a living factory that can produce functional proteins and next-generation biomaterials. The blend of high-throughput genomics, AI-driven design, and exact genome editing is overcoming previous technical barriers, opening new possibilities for manipulating this organism. Looking to the future, the success of these technologies will rely on a balanced approach that encourages innovation while ensuring safety. As these milestones are reached, CRISPR-edited silkworms hold great promise as valuable models for basic science and are poised to contribute significantly to a sustainable 21st-century bioeconomy (Figure 2).

### 5.1. Development of CRISPR Future Technologies in Silkworm

The CRISPR system has truly transformed genome editing in silkworms, opening new possibilities. While there is still much to explore, future efforts will aim to make editing more efficient, minimize off-target effects, and broaden the range of editing techniques, all while better aligning with silkworms’ unique biology. These innovative advancements are set to accelerate progress in functional genomics, molecular breeding, and sericultural biotechnology, benefiting the entire field. Furthermore, incorporating advanced high-throughput screening platforms, like multiplexed sgRNA libraries, will make it easier and faster to test and discover promising sgRNAs. This streamlined approach will accelerate in vivo genome-scale studies, opening new doors for research [23].

Using deep learning methods for CRISPR off-target prediction shows promising results. When we incorporate experimentally validated off-target data and consider important factors such as sequence mismatches, bulges, PAM context, and chromatin accessibility, it can significantly strengthen models and improve their performance across different scenarios [175]. Building and training specialized AI models for genomic analysis can significantly advance research in the silkworm. With these tools, we can better screen and assess sgRNA risks, leading to more accurate control over complex traits such as silk production. This also helps minimize accidental edits that might interfere with important metabolic process pathways.

### 5.2. CRISPR-Mediated Modification of Synthetic Biology Chassis

The silk gland of the domesticated silkworm provides an excellent foundation for synthetic biology, offering a scalable platform for protein production and biomaterial development. With the help of CRISPR technologies, future research can carefully modify this organ to overcome its natural limitations and connect fundamental biology with industrial uses in sustainable biomanufacturing. The silk gland offers many benefits as a synthetic biology platform; it can produce large amounts of silk proteins and is especially well-suited for high-level expression of complex molecules. This makes it a wonderful choice for creating therapeutic proteins and innovative materials [9,10].

CRISPR systems also serve as powerful tools for the engineering of efficient production hosts, enabling the reconstruction of biological processes and the optimized expression of heterologous genes. Malfunctioning the molecular processes governing high-level endogenous protein expression, such as Fib-H, facilitates the elucidation of the roles of various transcription factors, epigenetic modifications, and noncoding RNAs. Such research results provide guidance for developing improved strategies to increase production [78,145]. For exogenous proteins, using CRISPR-based optimization strategies—like multi-locus knock-in and promoter engineering—can help improve how well these proteins integrate, decrease mosaicism, and strengthen genetic stability [18,63].

### 5.3. From Silkworm to Lepidopteran Pest Control

The silkworm, as the main genetic model for Lepidoptera, with a well-developed CRISPR toolkit, is an incredibly valuable platform for testing genetic pest control methods. It also sparks innovation and supports new techniques. Thanks to its easy rearing, inability to fly, and great flexibility for genetic changes, it allows for quick development of high-risk strategies like gene drives in a safe and controlled way.

The silkworm has long been a key subject in the study of how insects regulate their sex. Going back to the 1950s, researchers developed a balanced lethal system using non-meiotic division, which allowed silkworms to develop parthenogenetically [176]. CRISPR technology has significantly enhanced the accuracy of this traditional approach. Recent studies have successfully used CRISPR to target the sex-determination genes *Bmdsx* and *BmMasc*, leading to outcomes like sexual reversal or female-specific lethality [44,177,178]. These systems can be directly adapted to develop sex-ratio manipulation or sterilization tools for lepidopteran pests, such as corn borer or diamondback moth, to help control their populations.

At the same time, the whole-genome CRISPR library of silkworm creates an opportunity to enhance its ability for target discovery capacity [23]. High-throughput screening, conducted at both cellular and organismal levels, has already opened the door to discovering many genes associated with viral resistance, environmental stress, and pesticides. This progress offers opportunities to develop broad-spectrum pest control solutions that can be applied effectively [72,171].

Finally, it should be noted that although the silkworm provides a powerful model for innovation, translating its outcomes to agricultural pests requires cautious, stepwise validation strategies. For example, at the technical level, it is necessary to consider the high genetic diversity of lepidopteran pests in the field, which may lead to resistance to drive systems through target-site mutations; at the ecological level, rigorous risk assessment of potential off-target effects and their ecological consequences is required; at the regulatory level, the release of such genetic control factors should occur under the supervision of an appropriate regulatory framework.

### 5.4. High-Throughput Functional Genomics and the Pangenome Era

The future of functional genomics involves using high-throughput CRISPR screens to explore these data. Genome-wide sgRNA libraries that target thousands of genes are now being used to discover the key factors that influence cell survival, resistance to viruses, and how cells respond to environmental toxins [171,179,180]. While cell-based screens have given some valuable early insights, the field is quickly moving towards high-throughput in vivo screening. Instead of relying on labor-intensive methods, the emphasis is now on building individual mutation libraries. This approach makes it possible to study many genes at once within a living organism, pointing towards a future for functional genomics [181].

Moreover, deep learning models that are trained on pangenome data hold potential to predict the causal variants behind complex traits like cocoon weight and silk fineness [1]. Testing these predictions with techniques like CRISPR-based base editing or prime editing can greatly speed up the breeding process, thereby enabling faster adaptation of silkworm strains to industrial needs.

### 5.5. The Convergence of AI and CRISPR in Sericulture

The integration of AI and CRISPR technology holds significant promise for the ongoing evolution of sericultural science. Deep learning algorithms are changing the way experiments are designed by accurately predicting sgRNA efficiency and off-target effects. Models like CRISPR-Net and various CNN-based architectures trained specifically on genomic data now include factors like chromatin accessibility and sequence context [175,182]. This progress is making gene-editing experiments more successful and reliable. Beyond guide design, generative AI models like ProteinMPNN and AlphaFold are being employed for the de novo design of novel silk proteins [183]. Researchers imagine sequences that do not naturally occur but are designed to fold into stable β-sheet structures. This creative approach allows them to craft synthetic spidroins with mechanical properties that exceed those of natural proteins [184].

### 5.6. Ecological Safety, Ethics, and Regulatory Landscapes

As genome-edited silkworms move closer to commercial use, it is important to develop robust ecological risk assessments and clear regulatory frameworks. The main ecological concern is the potential for gene transfer to their wild relatives, such as *B. mandarina*. Such introgression could theoretically alter the genetic integrity of wild populations or introduce traits with unforeseen ecological consequences. Even though domestication has made *B. mori* flightless and reliant on humans [185], providing a foundational level of physical containment, this passive barrier may be insufficient for large-scale open rearing scenarios. To mitigate these risks, future strain development should incorporate active molecular containment strategies. Approaches such as inducible lethality systems can serve as a “kill switch” to prevent survival outside controlled environments [186]. Finally, future research should also carefully measure the fitness costs of transgenes in hybrid backgrounds. This will help ensure that any escaped alleles are naturally removed from wild populations, contributing to environmental safety.

## Data Availability

No new data were created or analyzed in this study.

## Abbreviations

The following abbreviations are used in this manuscript:

ZFNs	Zinc Finger Nucleases
TALENs	Transcription Activator-Like Effector Nucleases
CRISPR	Clustered Regularly Interspaced Short Palindromic Repeats
NHEJ	Non-Homologous End Joining
RVD	Repeat-Variable Diresidue
DSBs	DNA Double-Strand Breaks
HDR	Homology-Directed Repair
PAM	Protospacer Adjacent Motif
BE3	Base Editor 3
MMEJ	Microhomology-Mediated End-Joining
ssODN	Single-Stranded Oligodeoxynucleotide
RNP	Ribonucleoprotein
CGA	Chlorogenic Acid
ISQ	Isoquercitrin
Orco	Odorant Receptor Co-Receptor
20E	20-hydroxyecdysone
JH	Juvenile Hormone
T2T	Telomere-to-Telomere
CNN	Convolutional Neural Network
AI	Artificial Intelligence
